# Energy consumption of standard and contrast-enhanced mammography: a step towards sustainable breast imaging

**DOI:** 10.1007/s00330-026-12373-2

**Published:** 2026-02-11

**Authors:** Gabriele Rossini, Kieran Lockey, Maxime Rokoszak, Marcella Pasculli, Carla Benstead, Fleur Kilburn-Toppin, Richard Hales, Jan Vosshenrich, Ferdia A. Gallagher, Manuel Signorini, Elisabetta Giannotti

**Affiliations:** 1 Department of Breast Imaging, Fondazione IRCCS Istituto Nazionale dei Tumori, University of Milan, Milan, Italy; 2 Capital, Estates & Facilities Management Department, Addenbrooke’s Hospital, Cambridge University Hospitals NHS Foundation Trust, Cambridge, UK; 3 Department of Radiological, Oncological and Pathological Sciences, Sapienza-University of Rome, Rome, Italy; 4 Cambridge Breast Unit, Addenbrooke’s Hospital, Cambridge University Hospitals NHS Foundation Trust, Cambridge, UK; 5 Department of Radiology, University Hospital Basel, Basel, Switzerland; 6 Imaging, Cambridge University Hospitals NHS Foundation Trust, Cambridge, UK; 7 Department of Radiology, University of Cambridge, Cambridge, UK; 8 Radiology Department, ULSS 5 Polesana, Rovigo, Italy

**Keywords:** Climate change, Radiology, Mammography, Carbon dioxide

## Abstract

**Objectives:**

To quantify and compare the energy consumption of standard digital mammography (DM) and contrast-enhanced mammography (CEM), assess differences across manufacturers, and identify strategies to improve energy efficiency.

**Materials and methods:**

This prospective study measured direct energy consumption from three mammography systems across two vendors in a tertiary breast care centre. In total, 193 examinations were analysed: 79 on Machine A, 92 on Machine B, and 22 CEM exams on Machine C. Minute-by-minute power monitoring provided net and gross energy per exam. A multivariable regression model adjusted for machine type, exam characteristics, and patient variables. Daily-level analyses evaluated baseload energy relative to workload, and annual energy use was estimated via Monte Carlo simulations.

**Results:**

Machine B consumed more net energy per exam but achieved the lowest gross energy use due to minimal standby power. Machine A showed lower net but higher gross energy, primarily from greater idle consumption. Machine C had comparable net energy to A but higher gross energy per exam as a consequence of fewer daily exams. Machine type was the dominant determinant of energy use, while exam type, breast thickness, and density had no significant impact. Higher daily exam volumes improved energy efficiency across all systems, particularly for C. Annual energy estimates ranged from ~1660 to 2300 kWh per machine, with B consistently most efficient.

**Conclusion:**

Mammography exhibits modest energy consumption, largely driven by standby operation rather than imaging activity. Vendor-specific differences exist, but DM and CEM show comparable net energy use.

**Key Points:**

***Question*** *Measure the energy use of standard digital and CEM, evaluate energy efficiency across vendors, and identify ways to reduce energy consumption*.

***Findings*** *Mammography uses relatively little energy, mostly during idle time; energy efficiency varies by manufacturer, and CEM does not increase net energy demand*.

***Clinical relevance*** *Optimizing scheduling appointments, powering down machines after hours, and considering vendor efficiency can significantly cut the environmental footprint of breast imaging without compromising patient care*.

**Graphical Abstract:**

**Figure:**
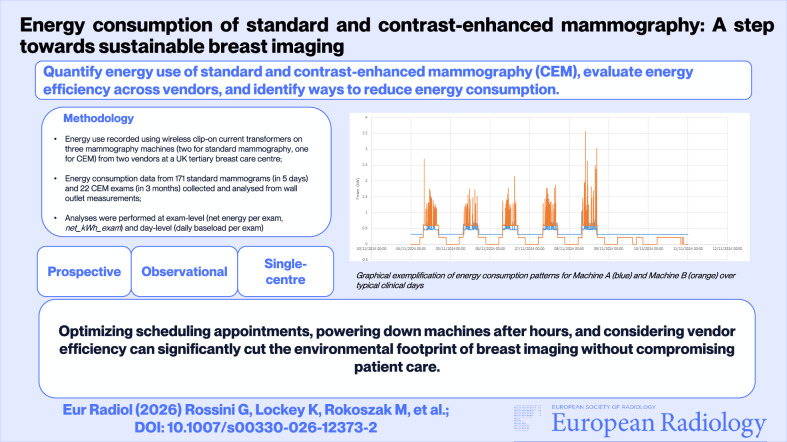

## Introduction

In recent years, climate change has emerged as a critical global health threat. It is estimated to cause 250,000 annual deaths by 2030 and attracts increasing awareness for numerous adverse effects [1, 2]. Paradoxically, healthcare itself is a significant contributor to climate change, ranking among the most carbon-intensive service sectors in industrialised nations [3–7]. The global healthcare system is estimated to account for roughly 4.5% of worldwide greenhouse gas (GHG) emissions [7], with different countries contributing to varying extents. The healthcare sector represents up to 8–10% of the national GHG footprint in the United States, and around 4% in the UK [3, 8]. If ranked as a country, global healthcare would be the fifth largest GHG emitter [4, 5]. Additionally, climate change-related problems, including air pollution, extreme weather and its consequences, infectious diseases, and mental illnesses, are in turn also likely to increase healthcare services demand, particularly among more vulnerable populations, thereby increasing health inequities [3, 4, 8, 9].

As research regarding the environmental impact of healthcare continues to expand, radiology has emerged as a significant contributor to the sector’s carbon footprint [1]. This is due to the high energy-consuming nature of imaging technologies, waste production and transport of staff and patients, with radiology alone estimated to account for approximately 1% of global GHG emissions [5, 8, 10]. There is growing interest in orienting healthcare practices towards their core mission of minimising harm and safeguarding public health, while addressing the urgent need for sustainability [4, 11]; in our field, this is termed “green radiology”. Identifying effective strategies to lessen environmental impact is both a responsibility and an opportunity for innovation [4, 12, 13].

Several studies have investigated the environmental impact of radiology techniques, demonstrating that magnetic resonance imaging (MRI) and computed tomography (CT) are energy-intensive. They account for a considerable amount of the total energy consumption and carbon emissions within hospitals [4, 5, 11, 12]. Most of the energy consumption often occurs non-productively while imaging equipment is not in use [14]. To date, however, data on mammography, the cornerstone of breast cancer screening worldwide, are lacking. In the UK, over 2 million women undergo mammographic screening every year [15] and, in the United States of America (USA), the total mammography procedures reported per annum are more than 42 million [16]. These figures highlight the significant role breast units play in energy consumption and emphasise the need to identify and apply energy-saving strategies for mammograms and their impact on emission levels. Additionally, contrast-enhanced mammography (CEM), has emerged as a viable alternative to MRI [17] and is expanding in clinical practice.

This study aims to fill the gap in the literature by directly quantifying the energy consumption of both standard digital mammography (DM) and CEM. We also assess energy efficiency differences between manufacturers and discuss potential energy-saving strategies.

## Materials and methods

### Study setting and data collection

This study prospectively collected energy consumption data from three mammography machine units of two different vendors, at a tertiary breast care centre.

For DM, data were collected from 171 consecutive exams performed from November 4th, 2024, to November 8th, 2024: 79 exams acquired on machine A (Senographe Pristina, GE Healthcare) and 92 on machine B (Selenia Dimension, Hologic), during clinical routine. At a single-scan level, both groups included standard 2D bilateral and unilateral mammograms [243/284 scans (85.6%) on machine A; 276/310 (89.0%) on machine B], unilateral tomosynthesis [14/284 scans (4.9%) on machine A; 18/310 (5.8%) on machine B) and magnification views [27/284 scans (9.5%) on machine A; 16/310 (5.2%) on machine B].

For CEM, data were collected from 22 consecutive exams performed from December 1st, 2024, to February 28th, 2025, on machine C (Senographe Pristina, GE Healthcare; same vendor as machine A). The CEM protocol consisted of 17 cases (77.3%) with four dual-energy acquisitions (bilateral craniocaudal and medio-lateral oblique projections), and, in 5 cases (22.7%), two additional delayed lateral projections were performed.

#### Reader assessment and recorded variables

Three radiologists (G.R., E.G. and M.P.) with 3–15 years of experience in breast imaging, alongside a lead quality assurer radiographer (C.B.), recorded the number of examinations and exam starting time for each day. Breast density category (A, B, C or D) was assessed by consensus and assigned according to the American College of Radiology (ACR) BI-RADS® classification [18]. Breast thickness was recorded as automatically measured by the machine in the right craniocaudal projection. Age was not systematically collected, as this was a service evaluation, but patients spanned the typical screening and diagnostic age ranges.

### Energy measurement

Energy usage was measured at the wall outlet for each system. Wireless current transformers were attached to the power supply of each machine to monitor power consumption at one-minute intervals. The daily total energy usage was calculated and correlated with the exam durations, which allowed for the calculation of energy consumption per exam.

**Fig. 1:**
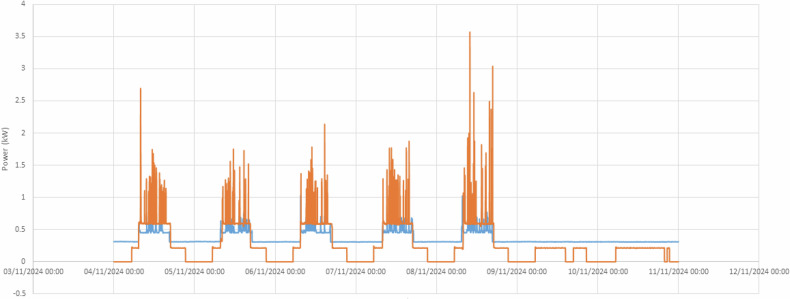
Graphical exemplification of energy consumption patterns for Machine A (blue) and Machine B (orange) over typical clinical days. The graph illustrates the distinct energy spikes associated with active imaging acquisition, as well as the differences in baseline (idle) energy consumption between the two systems. Machine A shows higher overall energy use and more pronounced out-of-hours energy consumption. In contrast, Machine B demonstrates a lower baseline but consumes more energy per individual exam, indicating higher energy use during active operation. These trends highlight opportunities for targeted energy-saving strategies tailored to each machine’s usage profile

Power spikes in machine consumption (Fig. 1) were matched with the number and times of exams for the day, as recorded. The baseline power was first identified. When usage exceeded baseline (e.g., peaking at ~0.75 kW for several minutes), it was converted to Kilowatt-hour (kWh) by dividing by 60 and summing the results for each minute to determine the net energy per exam. Daily total net energy was then obtained by summing the net energy of all exams for that day. Daily baseload, which represents the energy required for the machine to be on standby, was estimated as the difference between the machine’s daily total energy and the daily total net energy. Dividing this baseload by the number of exams performed that day gave a baseload per exam, which was added to the net exam energy to calculate the gross energy per exam.

### Statistical analysis

Analyses were performed at exam-level (net energy per exam, *net_kWh_exam*) and day-level (daily baseload per exam). Gross energy per exam was defined as net energy plus the apportioned baseload. Global comparisons across machines (A, B, C) were conducted using one-way ANOVA or Kruskal–Wallis tests with multiplicity-adjusted pairwise comparisons; effect sizes (η², ε², Cramér’s V, or *r* for two-group contrasts) are reported. Categorical variables were compared using χ² tests (or Fisher’s exact test when expected counts were small).

Outliers were identified during preliminary quality control and excluded prior to analysis. A multivariable linear regression model adjusted for machine, exam type (2D, tomosynthesis, magnification), density (ACR A–D), compressed breast thickness, and exam duration, using heteroskedasticity-consistent errors. Prespecified subgroup analyses were performed by density (A + B vs C + D), breast thickness (below/above median), and exam type.

At the day level, baseload per exam was modelled as a function of daily workload (number of exams/day) and machine, including interaction terms. Robustness of estimates was assessed with cluster bootstrapping (2000 replicates). Bootstrap confidence intervals were percentile-based; bootstrap *p*-values were derived from the empirical bootstrap distribution.

Annual energy consumption was estimated via Monte Carlo simulation (10,000 iterations) under observed- and equal-workload scenarios; results from parametric and non-parametric approaches were compared. For the parametric simulation, daily exam counts were sampled from Poisson distributions fitted to observed workloads, and baseload was sampled from Normal or Lognormal distributions depending on skewness; exam-level energy was resampled by bootstrapping.

Two-sided *p* < 0.05 was considered statistically significant, and 95% confidence intervals are reported throughout. All analyses were conducted in Python 3.10 (NumPy, pandas, statsmodels).

### Ethical approval

The study was registered as a service evaluation, and approval was obtained from the Quality and Safety Information System Clinical Audit Team (PRN13056; ID: 7056). As this was an observational study without any intervention, formal ethics approval and individual patient consent were not deemed necessary.

## Results

### Patients’ and exams’ characteristics

A total of 193 exams were included: 79 DM on machine A, 92 DM on machine B and 22 CEM on machine C.

Of the 79 exams of machine A, 77 were performed on female patients and 2 on males. The distribution of breast density categories was: A in 3/79 (3.8%), B in 58/79 (73.4%), C in 17/79 (21.5%), and D in 1/79 (1.3%). The 92 exams of machine B were performed on 92 female patients. The distribution of breast density categories was: A in 14/92 (15.2%), B in 39/92 (42.4%), C in 25/92 (27.2%), D in 14/92 (15.2%).

Descriptive statistics are reported in Table 1.

**Table 1:** Exam-level descriptive statistics for the three machines

Variable	Machine A (*n* = 79)	Machine B (*n *= 92)	Machine C (*n* = 22)
Net energy per exam (kWh)	0.050 ± 0.023; Median: 0.046 [0.034–0.066]; range: 0.01–0.15	0.091 ± 0.031; Median: 0.089 [0.070–0.110]; range: 0.03–0.15	0.051 ± 0.019; Median: 0.053 [0.044–0.062]; range: 0.03–0.10
Gross energy per exam (kWh)	0.574 ± 0.113; Median: 0.569 [0.491–0.650]; range: 0.33–0.87	0.411 ± 0.043; Median: 0.409 [0.375–0.445]; range: 0.32–0.47	4.40 ± 2.84; Median: 3.85 [3.80–8.20]; range: 1.22–9.38
Duration (min)	4.9 ± 2.2; Median: 4 [3–6]; range: 1–12	5.8 ± 2.3; Median: 5 [4–7]; range: 3–13	4.0 ± 1.6; Median: 4 [3–5]; range: 2–7
Thickness (mm)	53.5 ± 14.6; Median: 52 [41–62]; range: 33–82	55.3 ± 12.7; Median: 55 [47–64]; range: 40–71	51.6 ± 17.9; Median: 55 [34–68]; range: 34–80

Values are reported as mean ± standard deviation, median [interquartile range], and range

### Machine-level comparisons

Machine B showed higher net, but lower gross energy per exam compared with A, while C displayed a gross energy markedly higher than both due to a lower number of exams per day on which daily baseload was redistributed, despite similar net values. Exam duration and breast thickness were comparable across groups. Preliminary quality control identified two outliers in machine A (one exam with net 0.151 kWh and one lasting 12 min) and one outlier in B (duration 13 min); no outliers were found in C.

### Univariable analyses

Global comparisons confirmed significant differences across machines for net and gross energy per exam and exam duration, while breast thickness did not differ (*p* = 0.54). In pairwise analyses, machine B consumed significantly more net energy than both A and C, whereas A and C did not differ (*p* = 0.60). For gross energy, all pairwise comparisons were significant: A consumed more than B, and C was markedly higher than both A and B. Exam duration was significantly longer with B compared to A and C, with no significant difference between A and C (*p* = 0.18). Regarding categorical variables, exam type distribution did not differ significantly between machines A and B (*p* = 0.12), whereas breast density categories differed significantly across machines (*p* = 0.0007). These data are shown in Table 2.

**Table 2:** Global and pairwise comparisons of net energy, gross energy, exam duration, breast thickness, and categorical distributions across machines

Variable	Global test (*p*, effect size)	Significant pairwise differences (*p*-value)
Net energy per exam (kWh)	Kruskal–Wallis, *p* < 0.0001, ε² = 0.40	B > A (*p* < 0.0001); B > CEM (*p* < 0.0001)
Gross energy per exam (kWh)	Kruskal–Wallis, *p* < 0.0001, ε² = 0.80	A > B (*p* < 0.0001); CEM > A (*p* = 0.0001); CEM > B (*p* < 0.0001)
Duration (min)	Kruskal–Wallis, *p* = 0.0006, ε² = 0.07	B > A (*p* = 0.0066); B > CEM (*p* = 0.0034)
Thickness (mm)	ANOVA, *p* = 0.54, η² = 0.007	No significant differences
Exam type distribution	Chi-square, *p* = 0.12, *V* = 0.15	No significant differences
Density distribution	Chi-square, *p* = 0.0007, *V* = 0.25	Significant difference across machines

Only statistically significant pairwise results are reported; non-significant results are described in the text

### Multivariable analyses

In the multivariable regression model (adjusted *R*² = 0.88), machine B remained the strongest predictor of higher net energy per exam compared to A (β ≈ +0.030 kWh, 95% CI: 0.026–0.034, *p* < 0.001), while C also showed a smaller but significant increase vs A (β ≈ +0.0057 kWh, 95% CI: 0.004–0.007, *p* < 0.001). Clinical and technical covariates, including exam type, duration, breast thickness, and density, did not reach statistical significance, with small effect sizes and confidence intervals crossing zero. Cluster bootstrap resampling (2000 replicates) confirmed the stability of thickness- and density-related estimates, whereas the machine effects were less robust, with bootstrap medians close to zero and wide confidence intervals. Table 3 summarises these findings.

**Table 3:** Multivariable OLS regression of net energy per exam, adjusted for machine, exam type, duration, thickness, and breast density

Predictor	β (kWh)	95% CI	*p*-value	Standardized β	Interpretation
Machine B vs A	+0.030	0.026–0.034	< 0.001	0.90	Strong positive effect
Machine C vs A	+0.0057	0.004–0.007	< 0.001	0.17	Small positive effect
Exam type: tomosynthesis vs 2D	+0.0008	−0.003 to 0.005	0.72	0.02	Not significant
Exam type: magnification vs 2D	−0.0014	−0.017 to 0.014	0.86	−0.04	Not significant
Duration (min)	n.s.	–	–	–	Not significant
Thickness (mm)	n.s.	–	–	–	Not significant
Density A vs B, C vs B and D vs B	n.s.	–	–	–	Not significant

Coefficients (β) are reported with HC3 robust standard errors, 95% confidence intervals, *p*-values, and standardised β

*n.s.* not significant

### Day-level energy performance

At the day-level, five daily records were available for both machines A and B, and 17 for CEM. Results are summarised in Table 4.

**Table 4:** Day-level descriptive statistics and regression coefficients for baseload per exam

Machine	n_days	Exams/day (mean ± SD)	Baseload/exam (kWh, mean ± SD)	Model coefficients (bootstrap median [95% CI], p_boot)
A	5	15.8 ± 3.2	0.55 ± 0.13	n_exams: −0.032 [−0.042; −0.011], *p* = 0.002
B	5	18.4 ± 2.1	0.32 ± 0.03	B vs A: −0.139 [−0.187; −0.093], *p* = 0.004
C	17	1.8 ± 1.5	6.07 ± 2.56	n_exams: −1.39 [−4.29; −1.04], *p* < 0.001

For machines A and B, models included exam count and machine effect; for CEM, only exam count was fitted. Bootstrap cluster (2000 replicates) provided median coefficients, percentile-based 95% confidence intervals, and *p*-values

Machine B was consistently more efficient than A, showing a lower baseload per exam despite a slightly higher number of exams per day. Machine C exhibited much higher variability, with elevated baseload per exam due to the limited daily throughput, but improved efficiency with increasing daily exams.

Regression analyses confirmed that both a higher daily exam count (*p* = 0.016) and the use of machine B (*p* < 0.001) were significantly associated with lower baseload per exam. When interaction terms were included, the efficiency gain with increasing workload was steeper for A (slope –0.041 kWh/exam per additional exam/day) compared to B (–0.013 kWh/exam/day), but these interaction coefficients were not significant (all *p* > 0.2). Cluster bootstrap at the daily level confirmed the robustness of the main effects (exam count and machine B vs A), while for machine C a strongly negative slope (–1.39 kWh/exam/day, 95% CI: −4.29 to −1.04, p_boot < 0.001) indicated marked efficiency gains with higher throughput, albeit with wide confidence intervals due to the small sample of days.

### Simulation analyses

Monte Carlo simulations of annual energy consumption are summarised in Table 5. Under the observed-workload configuration, machine A consumed on average 1996, 2178, and 2269 kWh/year at 220, 240, and 250 working days, respectively. Machine B consistently showed lower consumption (1663, 1814, and 1889 kWh), with a gap of approximately 360–370 kWh/year compared to A at 240 days. Machine C showed intermediate values (1837, 2005, and 2088 kWh) but with greater variability due to limited daily throughput.

**Table 5:** Annual energy consumption estimated by Monte Carlo simulations under observed workload (A, B, C) and same-workload scenario (A vs B)

Machine	Scenario	220 days (kWh, 95% CI)	240 days (kWh, 95% CI)	250 days (kWh, 95% CI)
A	Observed workload	1996 (1991–2001)	2178 (2172–2183)	2269 (2263–2274)
B	Observed workload	1663 (1656–1670)	1814 (1806–1822)	1889 (1882–1897)
C	Observed workload	1837 (1825–1850)	2005 (1992–2018)	2088 (2075–2102)
A	Same workload	2025 (2018–2032)	2209 (2202–2216)	2301 (2294–2308)
B	Same workload	1663 (1650–1675)	1814 (1801–1827)	1889 (1882–1897)

Values are reported as mean with percentile-based 95% confidence intervals. Parametric simulations produced virtually identical results

In the same-workload scenario, using the exam count distribution of machine B, machine A remained less efficient (2025, 2209, and 2301 kWh) compared to B (1663, 1814, and 1889 kWh), confirming a technological difference independent of workload.

As a sensitivity analysis, parametric Monte Carlo simulations were performed by generating daily exam counts from Poisson distributions based on the observed workload and daily baseload values from Normal or Lognormal fits according to skewness, while exam-level energy was bootstrapped. Results were virtually identical to the non-parametric simulations (< 0.2 kWh difference in means and < 6 kWh in confidence intervals), supporting the robustness of the findings.

### Subgroup analyses

Robustness analyses confirmed that the higher net energy consumption of machine B compared to machine A was consistent across breast density and thickness strata. In non-dense breasts (ACR A + B), B consumed on average 0.091 ± 0.031 kWh vs 0.049 ± 0.024 kWh in A (β = +0.042, 95% CI: 0.032–0.052, *p* < 0.001); similar results were observed in dense breasts (ACR C + D: B 0.091 ± 0.030 vs A 0.052 ± 0.020 kWh; β = +0.040, 95% CI: 0.025–0.054, *p* < 0.001). C did not differ significantly from A in either subgroup.

When stratified by breast thickness at the median (55 mm), machine B again showed higher consumption in both thin (< 55 mm: B 0.086 ± 0.033 vs A 0.046 ± 0.020 kWh; β = +0.040, 95% CI: 0.029–0.051, *p* < 0.001) and thick (≥ 55 mm: B 0.097 ± 0.027 vs A 0.055 ± 0.026 kWh; β = +0.042, 95% CI: 0.031–0.054, *p* < 0.001) categories. Differences between C and A were not significant.

By exam type, machine A consumed more net energy for magnification views (0.091 ± 0.038 kWh) compared with 2D (0.048 ± 0.022 kWh, *p* = 0.02, *r* = 0.45), while tomosynthesis (0.056 ± 0.026 kWh) did not differ significantly. No differences by exam type were observed in machine B (2D 0.089 ± 0.031, tomosynthesis 0.090 ± 0.028, magnification 0.100 ± 0.020 kWh; global test *p* = 0.82).

## Discussion

This is the first study, to our knowledge, to systematically measure mammography energy use, showing it lies at the lowest end of imaging modalities while still offering scope for efficiency gains through improved power management and patient scheduling.

Mammography is among the most widely performed radiological examinations, primarily within breast cancer screening programs, reaching millions of women worldwide. Our study indicates that mammography (both standard and contrast-enhanced) does not incur high energy consumption and, moreover, most of it is unrelated to the number of examinations performed, but rather to keeping the machine operational. We also observed variability between vendors, both in overall and out-of-hours energy use patterns. Furthermore, no significant difference was observed between DM and CEM in terms of net energy consumption.

Environmental sustainability is receiving increasing attention within the scientific community and action is being taken: the NHS, for instance, became the first healthcare system to integrate net-zero emissions into legislation through the Health and Care Act of 2022, with a target to reduce emissions directly under its control by 80% by 2028 to 2032 compared with a 1990 baseline, and to reach net zero by 2040 [19]. Radiologists have a key role to play in this effort, as imaging departments contribute significantly to the environmental footprint of healthcare and hold potential for major positive change [20]. Imaging production accounts for a major portion of a radiology department’s carbon footprint. Recent studies have investigated the energy consumption of different imaging techniques. Merkle et al [21] demonstrated that an ultrasound machine consumes 2500 kWh annually, equal to about 50% of the yearly electricity consumption (and associated CO_2_ emissions) of one four-person household in Germany. Modern CT scanners consume between 20,000 and 30,000 kWh annually, equivalent to the consumption of 4–7 households. MRI consumption is even higher, with annual usage ranging from 80,000 to 170,000 kWh, equivalent to the needs of 16–34 households [21]. Strikingly, a large portion of this energy is consumed when the machines are in idle or non-productive states [12, 14, 20]. No prior studies, to our knowledge, have investigated mammography. From our study, we discovered that it lies at the lower end of the spectrum of imaging techniques’ energy consumption. Based on our analysis, the estimated annual energy consumption per mammography machine ranges from approximately 1660 to 2300 kWh. However, we observed significant variation in the energy profiles of the equipment, with certain manufacturers demonstrating greater energy efficiency, regardless of breast density, breast thickness and scan types.

In particular, machine B had the highest net, but the lowest gross energy per exam. Its increased demand for energy was balanced by much lower standby use, especially overnight. On the other hand, Machine A was more efficient during active imaging, but it had a higher constant baseload, which led to greater overall consumption. Machine C showed net energy values similar to A, indicating that dual-energy acquisitions in CEM do not require much more energy per exam than DM. However, its lower daily exam volume amplified the effects of the baseload, resulting in a higher gross energy per exam. At the daily level, across all systems, higher throughput consistently reduced the impact of idle consumption, with the greatest efficiency gain seen in machine C.

Multivariable modelling confirmed that machine type was the dominant factor in energy use, whereas exam type, breast density, and thickness had no significant effect. This highlights that energy consumption is largely driven by equipment design rather than patient or procedural factors. Stratified analyses reinforced these machine-level differences. Monte Carlo simulations further showed that machine B consistently consumed less than A, while machine C had intermediate but less stable performance. Overall, idle energy was a major contributor to total consumption, consistent with findings from CT and MRI studies, where non-productive states account for up to two-thirds of use [14].

Analysis of our data revealed actionable opportunities to further reduce unnecessary energy use, highlighting the role of scheduling and resource allocation in addition to equipment power management. In fact, beyond simply shutting down machines after hours, further savings are possible by improving patient scheduling. Consolidating appointments on fewer machines and maximising their daily usage can reduce idle energy consumption. This approach enhances energy efficiency and streamlines departmental processes and resource use [4].

Since mammography units are not emergency devices requiring constant operation, shutting them off after hours is a realistic strategy. Heye et al previously mentioned the benefits of turning off imaging equipment when not actively being used for diagnosis [22], estimating that up to 50% of medical imaging systems worldwide are not turned off during off-hours, representing huge and easily achievable energy and CO_2_ emissions reduction opportunities globally. Although the savings from a single mammography unit may appear negligible, the cumulative impact is substantial on a global scale. With millions of mammograms performed each year and more than 26,500 accredited units in the USA alone (MQSA National Statistics, Food and Drug Administration (FDA) [16]), the potential for worldwide reductions in energy use and costs is considerable. This idle consumption is avoidable: implementing consistent power-down practices could markedly lower energy demand, operational costs, and the overall environmental footprint of breast imaging.

Furthermore, CEM does not significantly increase net energy consumption per examination compared with standard DM. Beyond its established clinical value, CEM offers practical advantages over MRI, including lower costs, wider availability, shorter acquisition times, and improved patient comfort and tolerance [17]. Its relatively low energy use represents an additional benefit. Recent work [23] has proposed that carbon emissions in radiology should be subject to the same principles of justification and optimisation as radiation exposure, following the ALARA (As Low as Reasonably Achievable) framework. This underscores the importance of ensuring that clinically indicated imaging is also environmentally responsible. Where diagnostic accuracy is equivalent, the modality with the lower energy demand and environmental footprint should be preferred. Adopting this principle, first in breast imaging, and more broadly across radiology, can meaningfully reduce healthcare’s carbon footprint without compromising patient care.

This study has some limitations. First, this was an exploratory single-centre study. No sample size calculation was performed, and the analyses were not powered to detect small differences between groups. In particular, the number of CEM examinations was limited, which increases the risk of type II error. Results should be interpreted as descriptive and hypothesis-generating. Moreover, data were collected from only three machines and over a relatively short period, which may affect generalisability. Finally, the analysis considered only direct electricity consumption, without accounting for indirect emissions from imaging file storage, equipment production, transport, maintenance, disposal, or consumables. Future studies should involve a wider range of systems and institutions and include a larger number of examinations, should apply life cycle assessment for a more complete sustainability evaluation, and explore strategies such as intelligent power management or workflow redesign. Comparative studies across modalities, especially those used for related purposes (e.g., CEM vs MRI), could help create environmentally responsible imaging guidelines.

In conclusion, this study provides the first systematic evaluation of mammography energy use, showing it to be among the lowest of imaging modalities, yet still affected by avoidable inefficiencies, particularly during idle time. Optimising scheduling, powering down equipment after hours, and prioritising energy-efficient systems in procurement could yield substantial global savings. Notably, CEM consumes a similar amount of energy as DM and should be explored as a more sustainable alternative to MRI, highlighting the need to consider sustainability alongside clinical factors.

## Data Availability

Data available on request from the authors.

## Abbreviations

ACR: American College of Radiology
ALARA: As low as reasonably achievable
BI-RADS: Breast imaging reporting and data system
CEM: Contrast-enhanced mammography
CT: Computed tomography
DM: Digital mammography
FDA: Food and Drug Administration
GHG: Greenhouse gas
kgCO_2_e: Kilograms of carbon dioxide equivalent
kW: Kilowatt
kWh: Kilowatt-hour
MQSA: Mammography quality standards act
MRI: Magnetic resonance imaging
UK: United Kingdom
USA: United States of America
